# A Low-Power High-Sensitivity Photocurrent Sensory Circuit with Capacitive Feedback Transimpedance for Photoplethysmography Sensing

**DOI:** 10.3390/s24134097

**Published:** 2024-06-24

**Authors:** Neethu Mohan, Falah Awwad, Nabil Albastaki, Mohamed Atef

**Affiliations:** Electrical and Communication Engineering Department, United Arab Emirates University, Al Ain 15551, United Arab Emirates

**Keywords:** transimpedance amplifier, PPG sensor, optical receiver, automatic light control

## Abstract

This study presents an integrated analog front-end (AFE) tailored for photoplethysmography (PPG) sensing. The AFE module introduces a novel transimpedance amplifier (TIA) that incorporates capacitive feedback techniques alongside common drain feedback (CDF) TIA. The unique TIA topology achieves both high gain and high sensitivity while maintaining low power consumption. The resultant PPG sensor module demonstrates impressive specifications, including an input noise current of 4.81 pA/sqrt Hz, a transimpedance gain of 18.43 MΩ, and a power consumption of 68 µW. Furthermore, the sensory system integrates an LED driver featuring automatic light control (ALC), which dynamically adjusts the LED power based on the strength of the received signal. Employing 0.35 µm CMOS technology, the AFE implementation occupies a compact footprint of 1.98 mm × 2.475 mm.

## 1. Introduction

Optical sensing and its associated circuits have garnered significant attention within the realm of biomedical applications [1,2]. In contemporary medical and clinical practices, various health-monitoring devices utilizing photoplethysmography (PPG) have been developed. These devices offer a convenient means for physicians to measure blood pressure, monitor heart rate, and assess oxygen saturation levels without resorting to invasive measurement methods [1,3]. PPG sensory systems are highly versatile and find extensive applications in detecting a variety of diseases and physiological parameters. They are primarily used to monitor cardiovascular health [4], facilitating the detection of heart rate variability [5], arrhythmias [6], and peripheral vascular diseases [4]. By analyzing the pulsatile component of the PPG signal, clinicians can evaluate blood flow and oxygen saturation [5], which is essential for managing conditions such as chronic obstructive pulmonary disease and sleep apnea [7]. Additionally, PPG plays a significant role in monitoring stress and mental health by assessing changes in heart rate [7] and blood volume in response to stress [8]. It is also widely used in fitness and wearable devices to monitor physical activity and overall health metrics. Advanced applications of PPG include the early detection of diabetic vascular complications by glucose level monitoring [9] and continuous blood pressure monitoring [10], underscoring its importance as a tool in preventive healthcare and disease management. Thus, such optoelectronic systems combine optical and electronic components to detect, control, and manipulate light, playing a crucial role in various technological applications. One such application includes the in situ monitoring of vaginal pH using a bioresorbable fluorescence sensor [11].

A PPG sensory device typically comprises an optical transmitter and optical receiver [12,13], with the transimpedance amplifier (TIA) serving as a key component in the optical receiver. The transmitter subsystem consists of a light-emitting diode (LED) and an LED driver. The emitted light passes through the patient’s tissue, where it can be absorbed by the skin, bone, arterial blood, or venous blood. The intensity of the transmitted light varies depending on the volume changes in arterial and venous blood, which are influenced by cardiac rhythm [14]. The signal received by the optical receiver is the PPG input photocurrent signal, which has two parts—AC and DC. The AC component of the PPG signal is obtained depending upon the alterations occurring in blood volume, and this AC component, which is the main source of information, has a frequency similar to the heart rate (HR) (0.5 Hz to 5 Hz) [13]. The DC part of the received PPG signal remains, at most, a constant. Thus, this component of the PPG signal arises from factors depending on the signal-sensing direction, positioning of the finger, the wavelength of the light being used for transmission, the skin texture and color, and the variations in average blood volume [13]. The PPG(AC)/PPG(DC) ratio, which is defined as the perfusion index (PI), may vary from 0.1% to 3%, depending on the above-mentioned parameters [14]. PPG sensors detect blood volume changes by measuring variations in light absorption through tissue, with signal intensity influenced by ambient light, skin tone, tissue thickness, and sensor positioning [15]. An automatic light control (ALC) system adjusts the LED current to maintain consistent signal quality, ensuring a strong output at an acceptable LED current. ALC optimizes power consumption by reducing the LED current for strong signals. By adapting to individual user characteristics, ALC ensures consistent performance across diverse populations. For weak PPG output, the ALC module increases the LED current to produce higher optical power, maintaining a stronger output voltage. Conversely, for a too-high output signal level, the ALC module reduces the LED current to avoid distortion from saturation.

The conventional resistive feedback TIA (RF-TIA) faces bandwidth limitations imposed by the feedback resistor and its associated parasitic capacitance. To overcome this limitation, the capacitive feedback TIA (CF-TIA) presents itself as a viable alternative. The CF-TIA theoretically offers an equivalent gain and bandwidth performance compared to the RF-TIA [16,17]. Moreover, the CF-TIA mitigates thermal noise and addresses the challenge of integrating high resistance with CMOS technology, rendering it an advantageous choice for implementing low-noise CMOS TIAs [18,19]. CF-TIA falls short in terms of power efficiency when compared to advanced strategies like regulated cascode (RGC) or common gate (CG) TIAs [20,21]; however, CF-TIA has the capability to be designed to have high sensitivity with an input photocurrent range of nanoamperes [22]. An innovative approach using an inverter with active common-drain feedback TIA (ICDF-TIA) has demonstrated a higher gain compared to RGC-TIA at similar power levels [23]. Leveraging this, the proposed optical receiver in our study employs capacitive feedback with common-drain feedback TIA (CF-CDF-TIA) to achieve high gain and low noise while maintaining low power consumption. This configuration offers the advantage of striking an improved trade-off relationship between noise, gain, power, and bandwidth.

This paper introduces an optical receiver (AFE) utilizing capacitive feedback with common-drain feedback TIA (CF-CDF-TIA) to attain high gain and low noise while operating at low power consumption. The CF-CDF-TIA configuration proposed herein provides the advantage of enhancing the trade-off relationship between noise, gain, power, and bandwidth.

The rest of the paper is structured as follows: Section 2 introduces the proposed PPG sensory system and the analog front-end performance. Section 3 explains the LED driver and automatic light control (ALC) module. Section 4 presents the post-layout simulation results. Section 5 manifests a comparison of the proposed work with recently published works. The last section concludes the paper.

## 2. The Proposed PPG Sensory System

**Figure 1:**
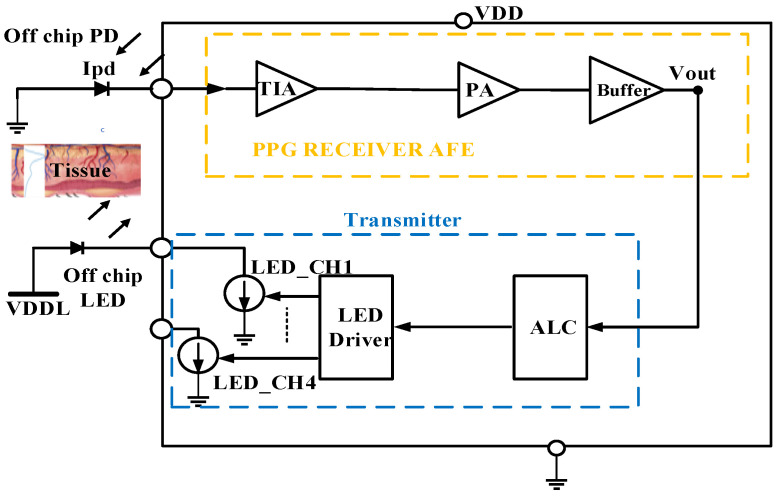
PPG sensor block diagram.

Figure 1 depicts the proposed PPG sensory system, which includes the integrated LED driver responsible for powering an external LED and emitting light for transmission.

This transmitted light penetrates the human tissue, where it is absorbed and subsequently reflected. The reflected light from the tissues is captured by an external photodiode (PD), converting it into a weak optical signal or photocurrent. Subsequently, the optical receiver’s amplification chain enhances the weak photocurrent. The signal strength undergoes conditioning through four comparators. The outputs of these comparators regulate the LED current level via feedback to the LED driver.

The automatic light control (ALC) module generates four distinct current levels, which are employed to modulate the LED driver, thereby adjusting the optical power of the transmitted LED signal—either increasing or decreasing it. This ALC functionality plays a pivotal role within the optical transceiver circuit, particularly as signal levels can fluctuate significantly due to various factors, such as distance, attenuation, absorption, and reflection. Through the accurate adjustment of the LED current, the ALC module ensures the preservation of signal integrity, thereby enhancing the system’s overall performance. Additionally, it effectively reduces LED power consumption when handling high input signal levels.

### 2.1. PPG Receiver Analog Front-End

**Figure 2:**
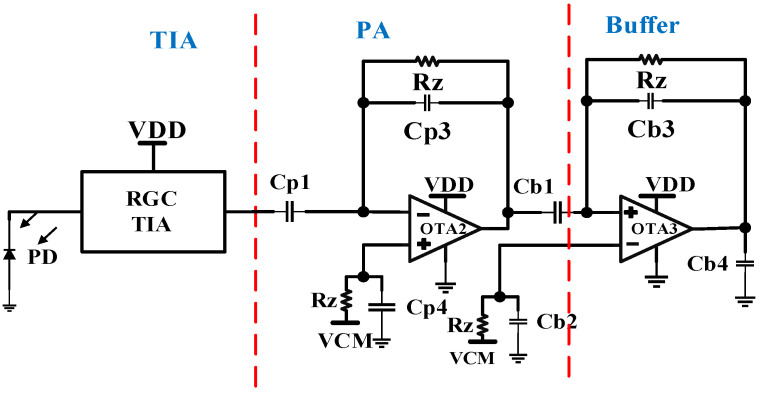
Analog front-end circuit for the PPG optical receiver.

The AFE illustrated in Figure 2 comprises three distinct stages. Firstly, the proposed capacitive feedback common drain feedback transimpedance amplifier (CF-CDF-TIA) serves as the initial stage. Following this is the second stage, which incorporates capacitive feedback post-amplifier (PA) to further amplify the signal. Within this module, the post-amplifier is realized using an operational transconductance amplifier (OTA) with capacitive feedback. Lastly, the final stage of this PPG optical receiver consists of an output buffer tasked with driving the capacitive input in the subsequent stage.

Table 1 above gives the design parameter values of the analog front-end circuit designed in this work.

### 2.2. Proposed CF-CDF-TIA Circuit

**Figure 3:**
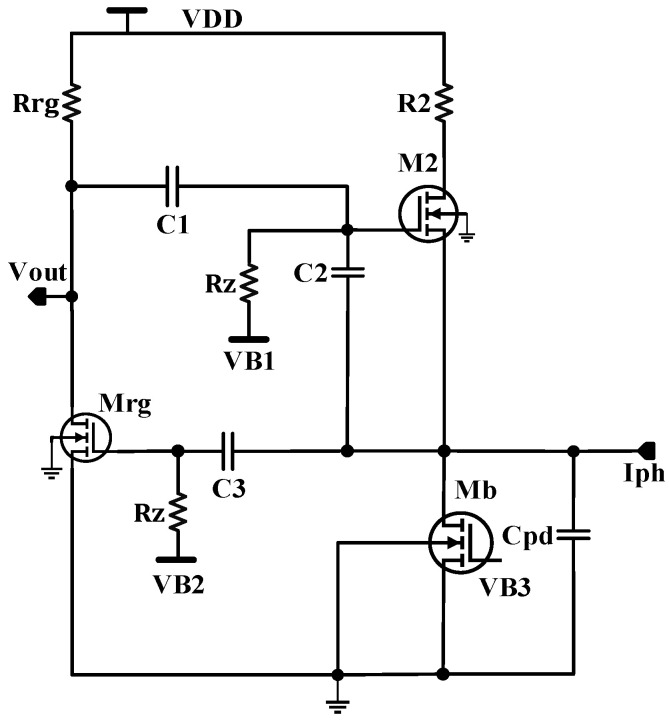
The proposed CF-CDF-TIA circuit.

Figure 3 illustrates the schematic of the proposed CF-CDF-TIA design. It features a common source voltage amplifier (Mrg, Rrg), which is responsible for regulating the gate of NMOS M2. This regulation is facilitated by connecting the capacitor C_1_ between the output node (Vout) of the common-source amplifier (Mrg, Rrg) and the gate of M2. Capacitor C_2_ establishes a connection between the gate of M2 and the input photocurrent (Iph). Together, C_1_ and C_2_ form a feedback path linking the input photocurrent to the output voltage. The input signal is AC coupled to Mrg via capacitor C_3_. Additionally, pseudo-resistors (Rz), connected in series with a constant biasing voltage source, are linked to the gates of both Mrg and M2. This biasing circuitry ensures the attainment of the requisite operating points. Both C_1_ and C_2_ play crucial roles in enhancing the gain of the CF-CDF-TIA compared to the conventional CDF-TIA discussed in reference [23], as described in Equation (11). This gain is contingent upon the chosen ratio of capacitor values for C_1_ and C_2_. From the small signal model, the transfer function of the transimpedance gain $Z_{TIA}\left(s\right)$ of the TIA can be derived, as given by Equation (1).

(1) $$Z_{TIA}\left(s\right)=\frac{-(a_{2}s^{2}+a_{1}s+a_{0})}{d_{3}s^{3}+d_{2}s^{2}+d_{1}s+d_{0}}$$

(2) $$d_{0}=g_{m2}R_{0}Rz+Rz+R_{0}$$

(3) $$d_{1}=g_{m2}R_{0}{Rz}^{2}C_{1}\left(g_{mrg}R_{rg}+1\right)+RzR_{rg}C_{1}\left(g_{m2}R_{0}+1\right)+R_{o}C_{1}\left(Rz+\;R_{rg}\right)+{Rz}^{2}\left(C_{1}+C_{2}\right)+R_{0}Rz\left(2C_{2}+C_{pd}\right)$$

(4) $$d_{2}=R_{rg}{Rz}^{2}C_{1}C_{2}\left(g_{mrg}R_{0}+1\right)+R_{0}{Rz}^{2}C_{1}\left(C_{2}+C_{pd}\right)+R_{0}{Rz}^{2}C_{2}C_{pd}+\;C_{1}R_{0}Rz\;Rrg\;(C_{pd}+{2C}_{2})$$

(5) $$d_{3}=C_{1}\;C_{2}\;C_{pd}\;R_{0}\;{Rz}^{2}\;Rrg$$

(6) $$a_{0}={-g}_{mrg}\;R_{0}Rz\;Rrg$$

(7) $$a_{1}=-Rrg\;g_{mrg}\;R_{0}\;{Rz}^{2}\;\left(C_{1}+C_{2}\right)$$

(8) $$a_{2}=R_{0}\;{Rz}^{2}\;Rrg\;C_{1}\;C_{2}$$

The transfer function above has two zeros and three poles. This transfer function is approximated by considering the following: Rz >> Ro >> $R_{rg},R_{2}$, and C_pd_ >> C_2_ >> C_1_. Ro is the internal output resistance of MOSFET Mb. By considering the dominant pole, the bandwidth can be calculated from the higher cut-off frequency $\omega_{H}$ and can be approximated as follows:

(9) $$BW{=f}_{H}\approx \frac{1}{2\pi }\frac{g_{m2}\;R_{0}\;C_{1}\left(R_{rg}\;g_{mrg}+1\right)}{{(R_{0}C}_{pd}\left(C_{1}+C_{2}\right)+C_{1}\;C_{2}\left(R_{0}+R_{rg}\right)}$$

The firs zero, $f_{Z1},$ determines the lower cut-off frequency and can be approximated as follows:

(10) $${f_{Z1}=f}_{L}\approx \frac{1}{2\pi }\frac{1}{\left(C_{1}+C_{2}\right)Rz}$$

The second zero position, $f_{Z2},$ is much higher than the dominant pole, which ensures the stability of TIA.

(11) $$f_{Z2}\approx \frac{1}{2\pi }\frac{g_{mrg}\left(C_{1}+C_{2}\right)}{C_{1}\;C_{2}}\gg f_{H}$$

The mid-band transimpedance gain can be approximated by the following relation:

(12) $$Z_{TIA}\;\approx \;\frac{-g_{mrg}\left(C_{1}+C_{2}\right)}{\frac{C_{1}+C_{2}}{R_{o}\;R_{rg}}+\frac{gm2\;C_{1}}{Rrg}+g_{m2}\;g_{mrg}\;C_{1}}\approx \frac{-C_{2}}{g_{m2}\;C_{1}}$$

The gain of the CF-CDF-TIA is augmented by the ratio of two capacitors, C_2_/C_1_, and does not introduce any additional noise in comparison to the ICDF-TIA lacking capacitive feedback [23]. This feature significantly reduces the total input noise current compared to that of the traditional RF-TIA. Moreover, the system’s gain is dictated by the ratio of the two capacitors, rendering the overall design less vulnerable to process variations. The transconductance g_m2_ in Equation (12) exhibits input signal dependency; as the input photocurrent increases, g_m2_ also increases, thereby inducing gain compression at higher input photocurrents. Capacitor C_3_ has a high pass filter effect, but it works with a high-valued pseudo resistor. The zero obtained from the effect of C_3_ and Rz can be expressed as

(13) $$f_{Z(C3)}\approx \frac{1}{2\pi }\frac{1}{C_{3}\;Rz}$$

C_3_ is 50 pF, and Rz is in the range of a few hundred giga ohm. Hence, the cut-off frequency is in the range of a few milli hertz. This explains the point that the effect of C_3_ can be neglected due to the cut-off frequency, the fact that its effect is in the range of a few milli hertz, and our system frequency of interest is 0.4 Hz.

### 2.3. Simulation Results for Capacitive Feedback CDF-TIA

**Figure 4:**
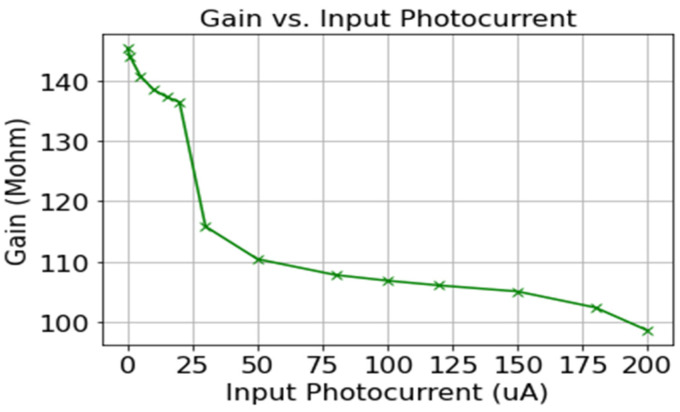
Plot showing input photocurrent vs. gain (dB).

This section presents the post-layout simulation results obtained for the proposed CF-CDF-TIA circuit. Table 2 outlines the design parameters utilized for the CF-CDF-TIA. Meanwhile, Table 3 displays the calculated and simulated values of bandwidth and transimpedance gain. The analysis indicates that as the input photocurrent rises, the gain diminishes. This can be clearly seen from Figure 4 below. Figure 4 shows the graph of gain vs. input photocurrent (from the range of 1 µA to 200 µA). Additionally, another observation drawn from this parametric analysis is that with the increase in the photocurrent from 1 nA to 200 µA, the bandwidth expands. There is a notable alignment between the analytical model calculations and simulation outcomes. Any calculation discrepancies are attributed to the oversight of parasitic capacitances and output resistances for the MOSFETs.

The derived mathematical model for the input-referred noise power density of the proposed CF-CDF-TIA is derived and given as follows:

(14) $${\bar{I}}_{n,in}^{2}=I_{n,MB}^{2}+I_{n,M2}^{2}+((I_{n,Rrg}^{2}+I_{n,Mrg}^{2}{)(\frac{j2\pi fC_{2}\left(C_{1}+C_{pd}\right)}{g_{mrg}\left(C_{1}+C_{2}\right)})}^{2})$$

**Figure 5:**
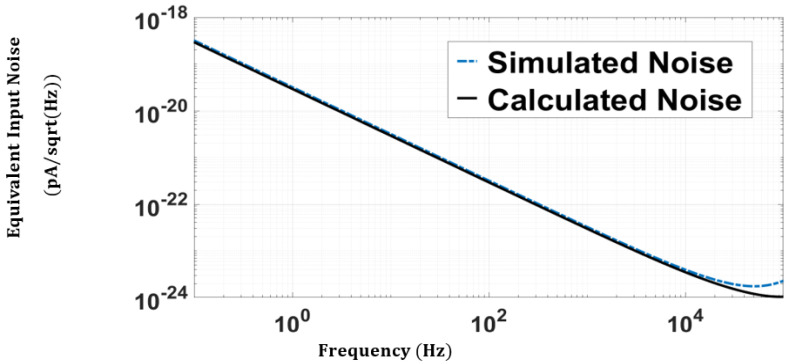
Simulated and calculated input-referred noise for CF-CDF-TIA.

Figure 5 compares the simulated and calculated input noise current for the CF-CDF-TIA. Figure 5 shows how closely the derived theoretical noise model fits with the simulated one. The calculated integrated noise current is equal to 6.93 × $10^{-10}$ A, and the simulated total input-referred noise current is 7.362 × $10^{-10}$ A.

### 2.4. Post-Amplifiers

The output of the TIA is in the range of a few millivolts. It is required to amplify this weak signal. The post-amplifier that follows the TIA performs this task by scaling the signal coming from the TIA. A post-amplifier with capacitive feedback is illustrated in Figure 2. The transfer function for the post-amplifier is given as follows:

(15) $$A_{o}\left(s\right)=\frac{-{sC}_{p1}R_{z}}{1+sC_{p3}R_{z}}$$

Here, $R_{z}$ is a pseudo resistor with a resistance value of a few hundreds of Giga ohms [10,13]. The use of $R_{z}$ in the OTA design of the post-amplifier helps the OTA input gate to obtain the required DC biasing.

The higher cut-off frequency for the post-amplifier design $\frac{1}{C_{p3}\;R_{z}}$ and for the model implemented in this paper, $F_{L},$ was designed to be less than 0.5 Hz. The OTA has a mid-band gain of 30 and consumes a biasing current of 1.567 µA. The output of the post-amplifier is given to a buffer amplifier, as can be seen in Figure 2. The buffer amplifier is crucial in PPG sensory systems for impedance matching, signal isolation, signal stabilization, and noise reduction. These functions are vital for ensuring accurate, reliable, and efficient signal processing. The transfer function for the buffer amplifier is given as follows:

(16) $$A_{b}\left(s\right)=\frac{-{sC}_{b1}R_{z}}{1+sC_{b3}R_{z}}$$

The higher cut-off frequency for the buffer design is $\frac{1}{C_{b3}\;R_{z}}$.

## 3. Automatic Light Control (ALC) and Led Driver

An ALC system automatically adjusts the LED current to maintain a constant output signal level despite variations in the input signal strength. The ALC module ensures that the output signal remains within the linear range even at wide variations in received optical power, thereby preventing distortion caused by signal overload or the excessive amplification of weak inputs. The ALC module continuously monitors the output voltage of the TIA. For weak input optical power, the ALC module increases the LED current to generate higher optical power to maintain a stronger output voltage. Conversely, for a strong input signal, the ALC module reduces the LED current to prevent signal distortion owing to clipping or saturation. The ALC module includes a control loop and feedback mechanisms that adjust the gain smoothly and rapidly for tracking changes in the input signal.

**Figure 6:**
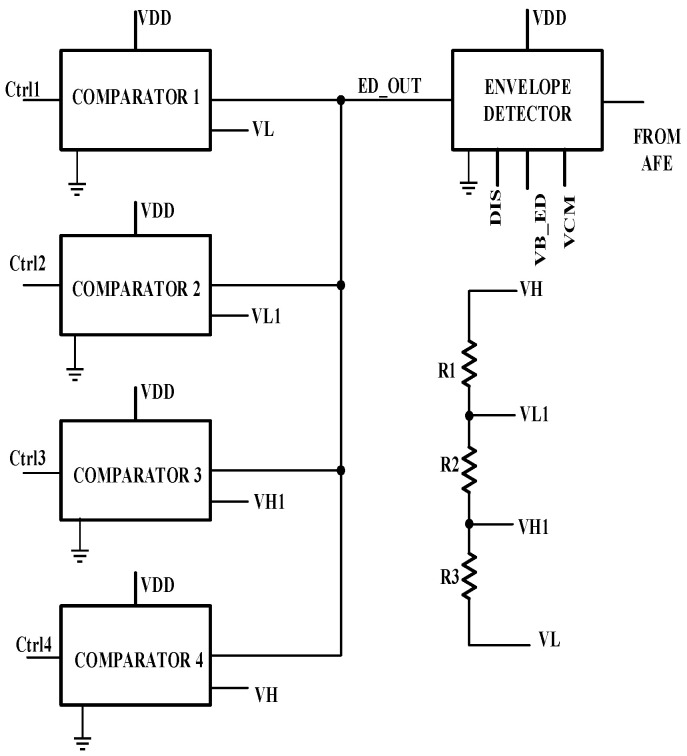
The automatic light control module.

Figure 6 shows the ALC module designed in this paper. It consists of an envelope detector and four comparators. The output of the amplifiers’ chain is provided as an input to the peak detector. The output of the peak detector is fed to four different comparators, which work at four different voltage levels between the maximum and minimum voltage levels detected at the output of the amplifier’s module. The ALC generates four different control signals (Ctrl1, Ctrl2, Ctrl3, Ctrl4), which control the LED driver’s current level. If the envelope detector output is below the threshold voltage of VDD/2 V, the amplitude is considered to be low, and the LED current is increased to the next level.

**Figure 7:**
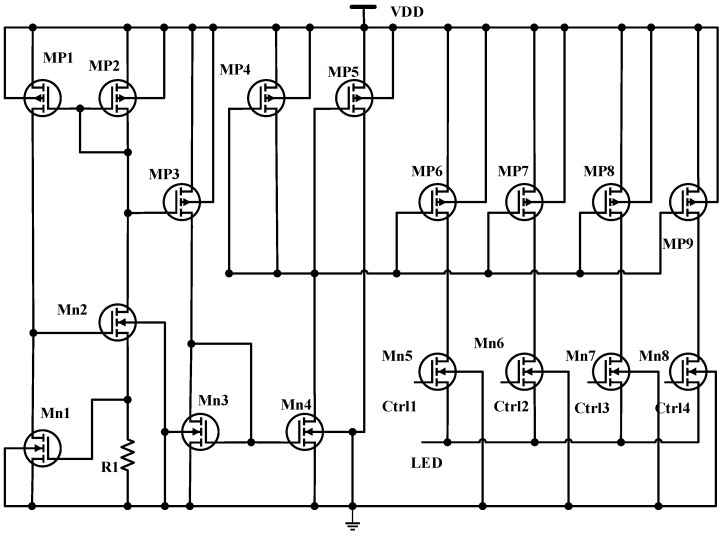
The LED driver circuit with ALC.

If the envelope detector values are above VDD/2 + 0.2 V, the signal amplitude is considered to be high; hence, the LED current is reduced by one step. The LED driver supplies the LED with the required current to emit enough optical power to have a high-quality PPG signal. Figure 7 below is the LED driver designed to have four channels. The four LED channel current levels are controlled through switching transistors $M_{n5},M_{n6},M_{n7},and\;M_{n8}$ using Ctrl1, Ctrl2, Ctrl3, and Ctrl4 from the ALC. The left side of the driver includes $M_{p1,2}$ and $M_{n1,2},$ and R_1_ provides the reference current, which is mirrored using $M_{n3,4}$ and $M_{p3,4,5}$ for the four channels $M_{p6,7,8,9}.$ Different mirroring ratios 2:4:8:16 are used to supply various LED current ranges from 400 μA to 11 mA for each channel. Table 4 below shows the LED driver’s MOSFET design parameters. The value of resistor R1 in the LED driver is 15 kΩ.

**Figure 8:**
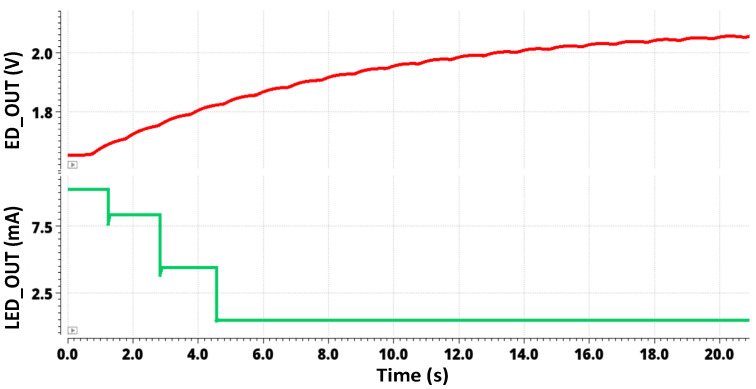
The envelope detector output and the LED driver circuit current for a 20 µA input photocurrent.

Figure 8 shows the transient simulation for a high-input photocurrent of 20 µA. The envelope detector output and the LED driver output for 20 µA are showcased in this figure. The output of the AFE contains both AC and DC components of the PPG signal. The envelope detector demodulates the signal, capturing the peaks and troughs of the pulsatile AC component, which represents varying blood volume. Initially, the signal is rectified by taking the absolute value of the AC component, making all parts of the waveform positive. This rectified signal is then passed through a low-pass filter, smoothing out rapid fluctuations and leaving a smooth envelope that corresponds to the peaks of the original signal. Figure 8 also indicates that the switching mechanism of the LED driver mentioned above reduces the LED current at high input photocurrent levels. Figure 8 indicates that different mirroring ratios 2:4:8:16 are used to supply various LED current ranges from 400 μA to 11 mA for each channel. The LED driver consumes a maximum power of 1.515 mW.

## 4. PPG Sensor Post-Layout Simulation

**Figure 9:**
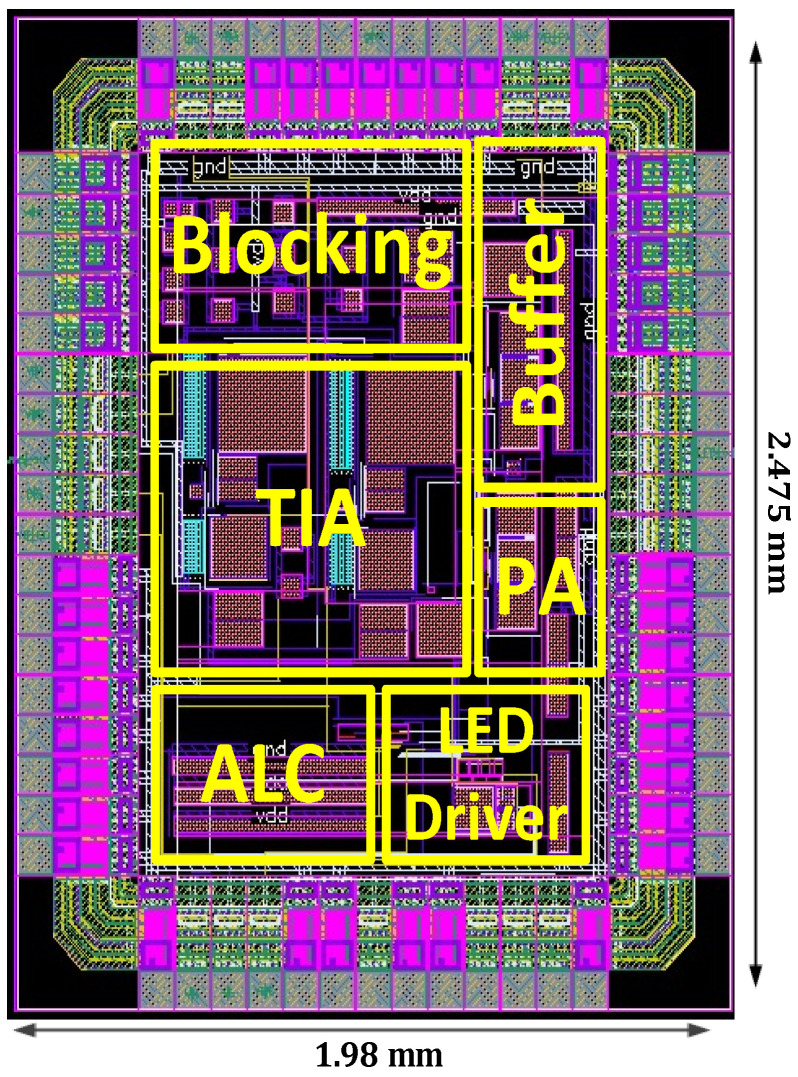
The PPG sensor layout.

**Figure 10:**
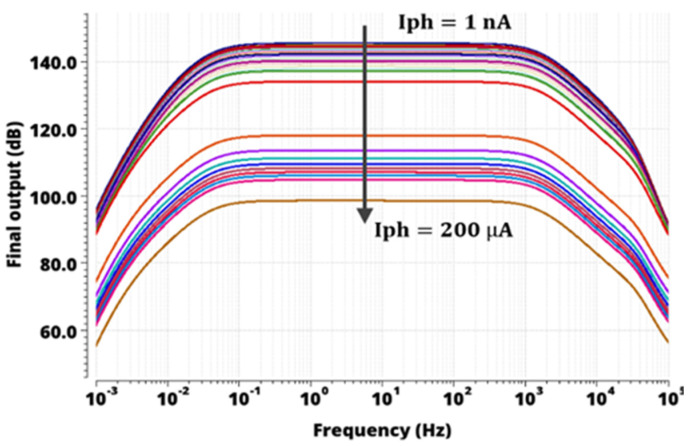
Frequency response for the proposed PPG sensory circuit for different photocurrents from 1 nA to 200 µA.

**Figure 11:**
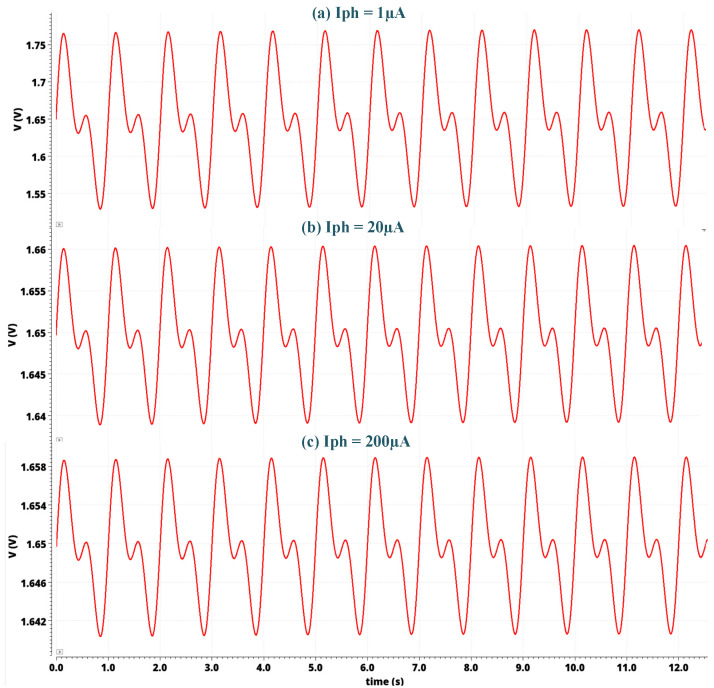
Output PPG voltage for input photocurrents of 1 µA and 200 µA, respectively.

**Figure 12:**
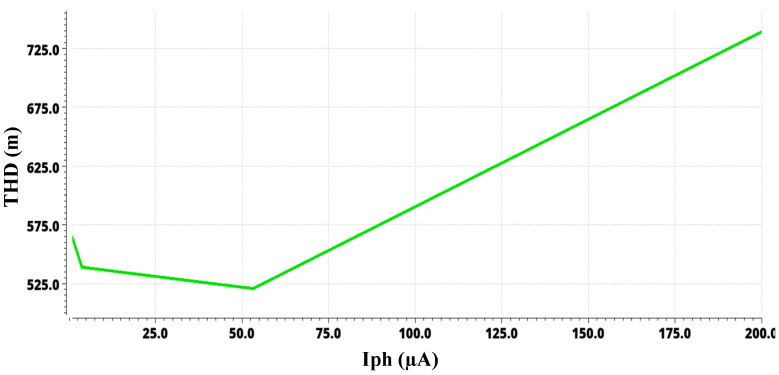
Simulation showing the total harmonic distortion for input photocurrent from 1 nA to 200 µA.

The complete system layout and the system simulations are implemented using AMS at 0.35 µm with CMOS Cadence Virtuoso technology. Figure 9 depicts the layout of the sensor module with a chip area of 1.98 mm × 2.475 mm. The post-layout frequency response simulation for different photocurrents from 1 nA to 200 µA is depicted in Figure 10. As the input photocurrent increases, the gain decreases. The output is amplified by 145.3 dB at a low input photocurrent while maintaining linearity at a high input photocurrent of 200 µA by reducing the gain to 98.73 dB. This gain reduction preserves the signal linearity at a high-input photocurrent. The overall bandwidth for the proposed system is 1.6 kHz for a low-input photocurrent. The overall bandwidth is limited by the post-amplifier and output buffer bandwidths, though not by the TIA bandwidth. Figure 11 illustrates the post-layout transient simulation for the PPG sensor module, highlighting that the ALC module ensures the output signal remains within the linear range despite wide variations in received optical power. This prevents distortion from signal overload or the excessive amplification of weak inputs. As shown in Figure 11, the PPG sensor system maintains its shape even with a high input photocurrent of 200 µA. The transient analysis used input photocurrents of 1 µA, 20 µA, and 200 µA. Moreover, the perfusion index of all three outputs of the PPG sensory system is shown in Figure 11 for different input currents of 1 µA, 20 µA, and 200 µA is 1 %. Figure 12 further elaborates on this point by showing the total harmonic distortion (THD) for input photocurrents ranging from 1 nA to 200 µA, demonstrating that the THD remains very low, in the range of a few milli, even at 200 µA. A THD in this range signifies that the PPG sensor system has high signal linearity, meaning that the output signal closely replicates the input signal with minimal harmonic distortion. A low THD indicates that the system introduces very little noise and few artifacts into the signal, which is critical for medical devices where noise can obscure essential physiological information or lead to incorrect readings. With minimal harmonic distortion, the PPG system can detect subtle changes in physiological signals, enhancing the device’s sensitivity and responsiveness. This is especially beneficial for monitoring conditions that require detecting minor variations over time.

**Figure 13:**
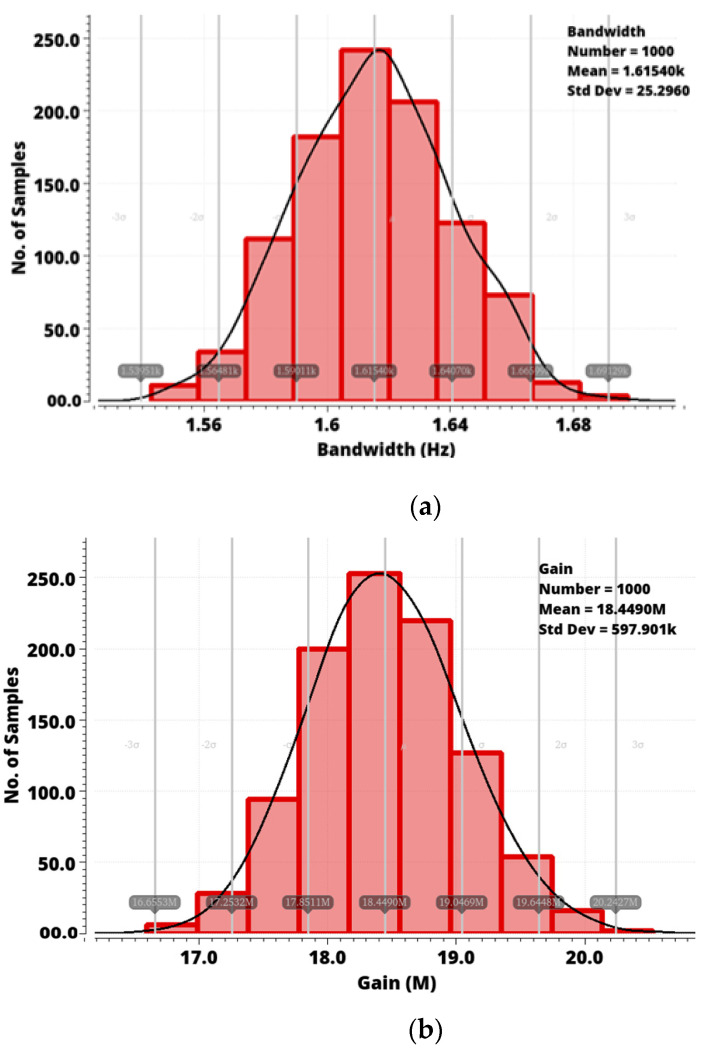
Monte–Carlo simulation for (**a**) bandwidth and (**b**) gain.

The effect of process variation on the proposed PPG sensor bandwidth and gain was analyzed using Monte–Carlo simulation. The bandwidth and gain changes were calculated for 1000 Monte–Carlo runs and are plotted in Figure 13a,b.

The Monte–Carlo simulation histogram for bandwidth has a mean bandwidth of 1.61 kHz and a standard deviation of 25.29 Hz. The histogram for gain variation depicts a mean gain of 18.44 MΩ and a standard deviation of 597.9 kΩ.

The investigation also involved approximately 36 corner simulations to analyze process–voltage–temperature (PVT) variations using corner analysis. PVT simulations entail executing simulations under diverse process corners to capture process variations, adjusting supply voltages, and considering varying temperature conditions. The findings presented in Table 5 offer valuable insights into the circuit’s performance across a spectrum of operational scenarios, empowering designers to make informed decisions and optimize designs for both reliability and performance. This comprehensive analysis encompasses the extremes of process variables as follows: worst noise (WN), worst speed (WS), temperature (−10 °C, 60 °C), and voltage supply (3.1 V, 3.5 V), as detailed in Table 5. By examining Table 5, it can be inferred that the maximum and minimum values of F_L_ and F_H_ result from PVT variations and ensure the preservation of an undistorted PPG signal, with its frequency components lying within the range of 0.1 Hz to 5 Hz (0.1 Hz < PPG signal frequency components < 5 Hz).

Table 6 elucidates the impact of $C_{pd}$ (external photodiode capacitance) on the overall system bandwidth, TIA (transimpedance amplifier) bandwidth, and the system’s input noise. It is evident from the table that an increase in photodiode capacitance leads to an augmentation in TIA bandwidth. However, variations in $C_{pd}$ values do not significantly affect the system’s bandwidth due to bandwidth constraints enforced in the second stage of the analog front end (AFE). Furthermore, an increase in $C_{pd}$ correlates with an elevation in system input noise, albeit the increment remains modest.

Table 7 presents the outcomes of the stability analysis conducted to assess the system’s stability. The stability simulations were executed across various values of $C_{pd}$. Observations from the table reveal that the lowest phase margin is 52.5, indicating the system’s stability even for photodiode capacitance fluctuation.

## 5. Comparison of Proposed Work Results

This section introduces a comparison of different performance parameters discussed in this work with state-of-the-art PPG sensors. Table 8 shows the comparison between state-of-the-art PPG sensors and the proposed one. In [24], H. Aminah et al. predict the sugar level using single-wavelength photoplethysmography. The system is accoutered using the 180 nm process. The system has an input-referred current noise of 7.3 pA/√Hz. In [13], L. Binghui et al. presents a PPG sensory system for continuous health monitoring. The PPG chip is fabricated using 350 nm standard CMOS technology. The average power consumption of the receiver analog front-end is 50.75 µW. The PPG sensor has an input noise current of 41.3 pA/√Hz and a gain of 11.9 MΩ. S. Wala et al. in [9] proposed a 180 nm process for photoplethysmography-based non-invasive glucose sensing. The work in [9] has an input photocurrent range of up to 65 µA. The system proposed in [5] by L. Qiuyang et al. implemented the 180 nm process. It is a PPG-based, non-invasive light-to-digital converter with a maximum gain of 4 MΩ.

The system proposed in [25] by M. Atef.et al. utilize a 350 nm CMOS technology process to design and implement a photoplethysmographic (PPG) sensor with an integrated photodiode and an automatic dimming control LED driver. The system has an appreciable gain of 17 MΩ but has a very high power consumption of 3.36 mW. The input photocurrent ranges up to 70 µA, which is much lower than that presented in this work. In addition, the input noise current is also at a large value of 35 pA/√Hz. The performance of the proposed sensor in this study is better than the state-of-the-art PPG sensors’ performance. The proposed CF-CDF-TIA succeeded in reaching high gain and low noise levels at low power consumption.

Table 9 below shows a state-of-the-art comparison of the capacitive feedback and common drain feedback TIA presented in this system. In [26], a 65 nm CMOS process is utilized to design a TIA with a gain of 52 dB and a high input-referred noise of 2.03 nA. The bandwidth is as high as 19.090 kHz, and TIA power consumption is 464.4 µW. The proposed TIA has a much higher gain, lower power consumption, and lower input-referred noise compared to [26]. The work in [27] designs an ultra-low power, high sensitivity PPG sensor based on an inverted cascode transimpedance amplifier using a 130 nm CMOS process. The design has an input-referred noise of 0.868 nA, which is higher than the noise of the proposed system. The TIA designed in [28] has a very low input-referred noise of 0.486 nA, but the power consumption by TIA is 158.8 µW. The proposed TIA has a comparatively lower power consumption of 46.76 µW. A miller-compensated inverter transimpedance amplifier for PPG sensing with a gain of 92.9 dB is presented in [29]. This system has a low power consumption of 3.86 µW and a low input-referred noise current of 81.77 pA, but the TIA has a much lower gain compared to the proposed TIA presented in this paper.

## 6. Conclusions

This paper presents a PPG sensory chip that has a high sensitivity and a low power. To attain a low power sensory system along with a high gain and low noise, a novel CF-CDF-TIA was designed using a common drain feedback TIA with capacitive feedback. An automatic light control loop was also integrated to reduce the LED current at a high input photocurrent. The validity of the new configuration has been confirmed through the remarkable consistency observed among the analysis and post-layout simulation results. The achieved high sensitivity and low power consumption can enable the proposed integrated PPG sensor to be used for wearable health monitoring systems.

## Figures and Tables

**Table 1:** Proposed analog front-end circuit design parameters.

VCM	C_p1_	C_p3_	C_p4_	C_b1_	C_b2_	C_b3_	C_b4_
1.65 V	30 pF	1 pF	30 pF	15 pF	15 pF	15 pF	10 pF

**Table 2:** Proposed TIA circuit design parameters.

R_rg_	R_1_	R_z_	R_o_	C_1_	C_2_	C_3_	V_B1_, V_B2_, V_B3_	L_Mrg_, L_b_, L_2_	W_Mrg_, W_b_, W_2_
165 kΩ	85 kΩ	A few GΩ to 1 TΩ	587.65 kΩ to 3.825 kΩ	1 pF	10 pF	50 pF	3 V, 2 V, 0.6 V	10 µ, 0.8 µ, 10 µ	1 µ, 1 µ, 4 µ

**Table 3:** Comparison of calculated and post-layout simulated results.

I_ph_	I_Mrg_, I_Mb_, I_M2_ (µA)	Simulated Gain (dB)	Calculated Gain (dB)	Simulated BW (kHz)	Calculated BW (kHz)
1 nA	11.65, 2.527, 2.528	115.82	117.46	13.94	13.49
1 µA	11.65, 2.515, 3.515	114.4	114.37	16.38	15.72
100 µA	11.65, −70.52, 29.48	78	81.6	760.2	627.07
200 µA	11.65, −157.4, 32.31	74.5	79.1	886.2	698.4

**Table 4:** Proposed LED driver circuit MOSFET design parameters.

MOSFET	M_p1_	M_p2_	M_p3_	M_p4_	M_p5_	M_p6_	M_p7_	M_p8_, M_p9_	M_n1_	M_n2_	M_n3_	M_n4_	M_n5_	M_n6_	M_n7_	M_n8_
Width (µm)	50	50	50	20	20	40	80	160	40	10	2	20	100	100	100	100
Length (µm)	1	1	1	1	1	1	1	1	1	1	1	1	0.35	0.35	0.35	0.35
Current	44.4 µA	42.76 µA	45.09 µA	453.2 µA	453.2 µA	1.949 pA	1.94 mA	3.92 mA	44.4 µA	42.76 µA	45.1 µA	453.2 µA	31.5 aA	1.94 mA	3.92 mA	3.92 mA

**Table 5:** PVT analysis.

		Gain (MΩ)	F_L_ (mHz)	F_H_ (kHz)
3.5 V, 60 °C	TM	15.26	37.15	1.458
	WN	15.26	39.8107	1.458
	WS	30.71	36.3078	0.7912
3.5 V, −10 °C	TM	24.38	40.738	1.878
	WN	24.38	39.8107	1.878
	WS	69.93	37.1535	1.017
3.1 V, 60 °C	TM	15.07	40.738	1.457
	WN	15.07	40.738	1.457
	WS	30.65	36.3078	0.7909
3.1 V, −10 °C	TM	15.9	45.708	1.883
	WN	15.9	42.6580	1.883
	WS	68.88	36.307	1.016

**Table 6:** Effect of photodiode capacitance on bandwidth and input noise.

$C_{pd}$ (pF)	TIA Bandwidth (kHz)	System Bandwidth (kHz)	System Input Noise (pA/√Hz)
5	74	1.63	7.72 × 10^−10^
10	47.5	1.629	7.92 × 10^−10^
15	34.9	1.628	8.2 × 10^−10^
20	27.59	1.626	8.535 × 10^−10^
25	22.74	1.624	8.94 × 10^−10^
30	19.34	1.622	9.4 × 10^−10^

**Table 7:** Effect of photodiode capacitance on phase margin.

$C_{pd}$ (pF)	Phase Margin
5	55.2°
20	52.6°
30	52.5°

**Table 8:** Comparison between the state of the art and the proposed work.

Reference Citation	CMOS Process	I (Noise)	Gain (MΩ)	$I_{ph}$ (µA)	System Power (µW)
[24]	180 nm	7.3 pA/√Hz	1	20	15
[13]	350 nm	11.9 pA/√Hz	12.5	24	50.75
[9]	180 nm	$9.4\;{pA}_{RMS}$ (for 10 Hz)	1	65	32
[5]	180 nm	5.7 pA/√Hz	4	50	89
[25]	350 nm	79 pA	17	70	3360
This work (post-layout simulation)	350 nm	0.1924 nA, 4.81 pA/√Hz	18.43	200	68

**Table 9:** Comparison between the state of the art and the proposed transimpedance amplifier.

Reference Citation	CMOS Process	Photodiode Capacitor ($C_{pd}$)	Input-Referred Noise Current	Gain (dB)	Bandwidth (Hz)	TIA Power (µW)
[26]	65 nm	NA	2.03 nA	52	19.090 k	464.4
[27]	130 nm	20 pF	0.868 nA	119.9	201 k	30
[28]	180 nm	NA	0.486 nA	107.95	10	158.8
[29]	180 nm	60 pF	81.77 pA	92.9	10 k	3.86
This work (post-layout simulation)	350 nm	20 pF	0.736 nA	115.82	13.94	46.76

## Data Availability

No new data were created or analyzed in this study. Data sharing is not applicable to this article.
